# Factors associated with the choice of plant and animal science as a career among technical and vocational education post-secondary graduates in Nepal

**DOI:** 10.3389/fsoc.2025.1585391

**Published:** 2025-09-02

**Authors:** Jay Chaurasia, Mahesh Jaishi, Santosh Khanal, Balika Poudel, Vivek Ghimirey, Nobel Acharya

**Affiliations:** ^1^Department of Agricultural Extension and Rural Sociology, Institute of Agriculture and Animal Sciences, Tribhuvan University, Kathmandu, Nepal; ^2^Department of Agronomy, Institute of Agriculture and Animal Sciences, Tribhuvan University, Kathmandu, Nepal; ^3^Department of Soil Science and Agri-engineering, Agriculture and Forestry University, Bharatpur, Nepal; ^4^Department of Agricultural Extension and Rural Sociology, Agriculture and Forestry University, Bharatpur, Nepal

**Keywords:** career preference, further education, maximum likelihood estimation, plant science, workplace

## Abstract

**Introduction:**

The present study was conducted in 2024 to analyze factors affecting the career preferences of post-secondary graduates in Nepal. The factors that impact the career decisions of secondary school agricultural graduates still need to be more adequately realized.

**Methods:**

In this context, the study was designed as an ex-post-facto research. Graduates of three secondary institutions in Sindhupalchok who participated in the Technical and Vocational Education (TVE) program responded to the questionnaire. Participants were asked to rate their sociodemographic information, preferred profession, and influences on their career decisions using a 5-point Likert scale for specific occupational and personal characteristics. Factor analysis was conducted on the variables using IBM SPSS Statistics 26 Version.

**Results:**

Cronbach's alpha coefficients (0.73) were calculated to estimate the reliability of the factor scores. In addition, exploratory factor analysis determined that the sample size selected for this study was sufficient (KMO value of 0.590 exceeded the minimum threshold of 0.50, and Bartlett's chi-square value of 619.447; *p* < 0.01). Five factors were identified by utilizing maximum likelihood methods with Promax rotation; collectively, these factors accounted for 59.56% of the total variance. Five determinants impacted the career preferences of the participants: personal characteristics, academic accomplishments, physical surroundings, social factors, and economic conditions. The findings indicated that foreign employment ranked highest among all professions with an overall priority of 0.044. Additionally, the findings unveiled that factors such as a conducive learning environment (4.45), workplace quality (4.22), job security (4.20), future employment prospects (3.99), and workplace (4.13) exerted the greatest impact on the career preferences of post-secondary graduates.

**Discussion:**

The expertise and commitment demonstrated by employees are highly valued by employers. The practical workshop procedures also fosters reflection on strategies to enhance creativity and genuine enthusiasm for the profession. A quality workplace encompasses components such as rewards, recognition, and a culture that strongly fosters individuals' professional growth.

**Conclusion:**

Through the early involvement of students in the process of selecting a career path, it is possible that enthusiasm and job satisfaction for that particular career preference could be enhanced.

## 1 Introduction

Agriculture contributes to the socioeconomic wellbeing of the people through food production and employment (Li et al., 2024). The Ministry of Agriculture Development focused enhancing agricultural education to boost agricultural productivity and alleviate poverty (MoAD, 2016). In 2013, under Nepal's School Sector Reform Plan (2009-15), the Ministry of Education (MoE) instituted a Technical and Vocational Education (TVE) program in secondary schools, encompassing grades 9 through 12 (MoE, 2016). The TVE program was introduced in 99 model schools nationwide in the piloting stage (MoECDC, 2015). As of 2021, the number of schools gaining approval to run TVE programs in secondary schools has reached 506, which includes 190 and 44 schools with plant science and animal science, respectively (MoEST, 2022). According to the Nepal Labor Force Survey (2018/19), 54,871 (51% of 1, 07, 411 students enrollment capacity) students have enrolled in 506 schools in Nepal. The enrollment capacity of students in TVE schools is 10,571; however, the actual enrollment is 6,918 students in agriculture as of 2022 (MoEST, 2022).

The Plant Science and Animal Science program seeks to engage students in higher education, enabling them to contribute to agricultural organizations and enhance the significance of local agricultural enterprises within the broader economy (MoAD, 2016; MoECDC, 2015). Agricultural education in public schools has a rich heritage of developing students' skills and providing the abilities needed for agricultural employment through classroom, laboratory instruction, and supervised experience (Minde et al., 2015). These individual skills and abilities help to be employed in teaching, civil services, entrepreneurship, agro-based private and public sector jobs, and so on (Rana et al., 2018; Adhikari, 2021).

Career selection entails pursuing future rewards that may be achieved at different times, such as accessing a chosen occupation, earning cash, gaining independence, and improving one's skills (Walker and Tracey, 2012). The career preferences of agricultural students are to be studied in response to the growing job prospects and economic incentives (Leavy and Hossain, 2014). Although there is a vast range of occupations to pick from, each individual has their preferences and dislikes regarding these career options (Epstein, 2022). However, as a result of the current circumstances, agricultural students are compelled to pursue professions such as administrative services and commercial banking, instead of teaching, research, and extension occupations (Dul et al., 2012). They also appear to have contrasting perspectives on occupations focused on rural areas, but their acquired skills might be effectively exploited for rural development (Van der Ploeg et al., 2017).

Choosing a suitable job path that aligns with one's future life trajectory is a crucial concern for both individuals and society as a whole it involves considering multiple criteria (Kossek et al., 2021). Due to the multiple criteria, the topic of career selection can be considered a problem of multi-criteria decision-making (Sriram et al., 2022). Career selection can be considered as the level of maturity or preparedness to commit to a career path (Kulcsár et al., 2020). A playful attitude necessitates a focus on the future, suggesting that persons who are future-oriented are more likely to have a deliberate approach to their job choices (Sakellariou and Vecchiato, 2022). To account for the fact that not all graduates pursue careers directly related to their chosen field of study, they need to possess transferable skill sets (Nägele and Stalder, 2017).

The government's decision to implement the TVE program as a separate stream will facilitate students in enhancing their skills, improving their proficiency, and effectively navigating their college experience (Joshi et al., 2021). The incorporation of plant science and animal science curricula in secondary schools aims to enhance the development of national human resources, hence bolstering agricultural and rural advancement in Nepal (Babu and Sah, 2019; Kurata et al., 2021). The agricultural education curricula should address the educational and career preparation needs of students who desire careers outside of school-based teaching (DiBenedetto et al., 2018; Okiror et al., 2017).

According to (Benitti 2012) findings, several variables can potentially introduce high school students to novel career prospects and areas of study that they may not have previously contemplated. These factors can also impact high school students' understanding of agriculture, their perceptions of the sector, and their level of interest in it (Jean-Philippe et al., 2017; Jones et al., 2017). Nonetheless, to our knowledge, there is an insufficient of empirical evidence that elucidates the current circumstances and career inclinations, as agriculture education has only been recently implemented in secondary schools. High school students require substantial professional guidance, particularly in job selection (Zafar, 2019). During this time, students are encouraged to prepare for their future meaningful careers (Stephen and Fru, 2023; Green et al., 2020). However, still, most students have to be encouraged to work with farmers and need to be devoted to agriculture-based occupations (Chaudhary and Pasa, 2015; Jjuuko et al., 2019).

The primary objective of this study was to assess career preferences and factors affecting the career preferences of agricultural graduates from TVE schools. This study will bridge the knowledge gap of the penchant for different careers and the professional fulfillment of secondary school graduates. The result of this study will help to understand the flows and activities of agricultural education and the different constraints and opportunities in reaching out to agricultural students. The present study hypothesized that TVE in secondary school affects the career preferences of graduates through their physical environment, social factors, and economic status. The study may offer insightful information about the preferred careers of agricultural secondary school students, particularly those engaged in Technical and Vocational Education (TVE) programs. This study will further the fundamental notion that students of all backgrounds and academic abilities can benefit from TVE's agriculture education program. The agriculture industry is crucial in many economies, like that of Sindhupalchok District. This study is significant for investigating methods to increase the pool of students who are equipped to pursue careers that entail creating solutions to tackle the issues of the 21st century. The following research questions were outlined to achieve the objectives of the study:

- What are the major career preferences of the TVE students after graduation?
- What are the factors affecting the career preferences of agricultural graduates from TVE schools?

## 2 Theoretical framework of the study

Several elements, including personal traits, academic background, and socioeconomic circumstances, influence the career preferences of senior high school students. These factors can have a direct or indirect impact on the career preferences of the respondents. Motivation is essentially the factor that ultimately influences an individual's decision-making process.

### 2.1 Holland's theory of career choice

In this study, Holland's theory of career choice was used as a theoretical lens to inform the variable of participant interest in career preferences. According to John Holland's Theory of career choice, careers are shaped by the relationship between individual personality and the surrounding environment (Šverko and Babarović, 2016). Holland's Theory of Career Choice also known as RIASEC theory suggests that people choose careers that allow them to interact with like-minded peers. They want situations that enable the utilization of their skills and abilities, facilitate the expression of their views and values, and present engaging challenges and roles (Ertmer and Ottenbreit-Leftwich, 2010). Behavior is influenced by the relation between personality and environment. Holland's theory (1966) of career choice posited that most of the people possesses one of the six fundamental personality traits that influences the career preferences (Xie et al., 2024). The following items are:

- Realistic: Realistic jobs sometimes entail labor tasks that encompass practical, hands-on challenges and resolutions. They frequently engage with flora, fauna, and tangible elements such as timber, implements, and machines. Numerous vocations necessitate outside work and entail minimal paperwork or collaboration with others (Brown and Lent, 2012).
- Investigative: Investigative professions sometimes involve conceptual engagement and demand considerable cognitive effort. They may entail investigating facts and resolving issues cognitively (Feldman et al., 2008).
- Artistic: Artistic professions often entail engagement with shapes, designs, and concepts. They frequently necessitate self-expression, and the tasks might be executed without adhering to a definitive set of guidelines (Nauta, 2010).
- Social: These jobs often entail engaging, interacting, and instructing individuals. These professions frequently include assisting or rendering services to others (Spokane et al., 2002).
- Enterprising: Entrepreneurial professions often comprise initiating and executing projects. These professions might include guiding individuals and making determinations. Occasionally, they necessitate risk-taking and frequently pertain to business matters (Brown and Lent, 2012).
- Conventional: These professions often entail adhering to established protocols and practices. These professions may have a greater emphasis on data and specifics rather than concepts. Typically, there exists a distinct hierarchy of power to adhere to Foutch et al. (2014).

### 2.2 Person-environment fit (P: E fit) theories

Each individual possesses a distinct combination of interests and abilities. Various occupations, along with their respective specialties, demand specific talents and personality traits. If an individual with specific interests and aptitudes can secure a job that aligns with their interests and utilizes their skills, they would probably be highly compatible with the occupation. Person: environment fit theories are essentially an approach to career coaching that focuses on matching individuals with careers that align well with their characteristics and the demands of the work environment. Frank Parsons outlined this procedure when founding a vocational guidance organization in Boston, Massachusetts (Parsons, 1909).

Roger (1952) also employed this method, utilizing a set of inquiries covering a wide range of topics such as intelligence, achievements, and personal situations, to direct his interviews with individuals seeking guidance on career choices. The ‘Seven Point Plan' he developed during the 1950s was extensively utilized to offer career recommendations to individuals who had recently completed their education.

Holland (1997) expanded upon the theory of Person-Environment Fit. Holland focused on the distinctions among individuals, rather than their commonalities. The author categorizes individuals into six distinct categories: realistic, investigative, artistic, sociable, enterprising, and conventional. It is acknowledged that these types will exhibit varying occupational interests.

### 2.3 Developmental career theories (DCTs)

Developmental theories of job choice acknowledge that the process of selecting and adapting to employment is ongoing, and utilize psychological principles to elucidate career development mechanisms. Donald Super is commonly linked to the progressive approach to career advising (Super, 1957). He delineates the progression of an individual's career through five distinct “life stages”: growth, exploration, establishing, maintenance, and decline. According to (Super 1957), when an individual matures, they form a perception of themselves, known as their “self-concept.” This self-concept may then be used to compare their image of themselves with their perception of someone in a particular profession.

## 3 Materials and methods

### 3.1 Selection of the study site

**Figure 1:**
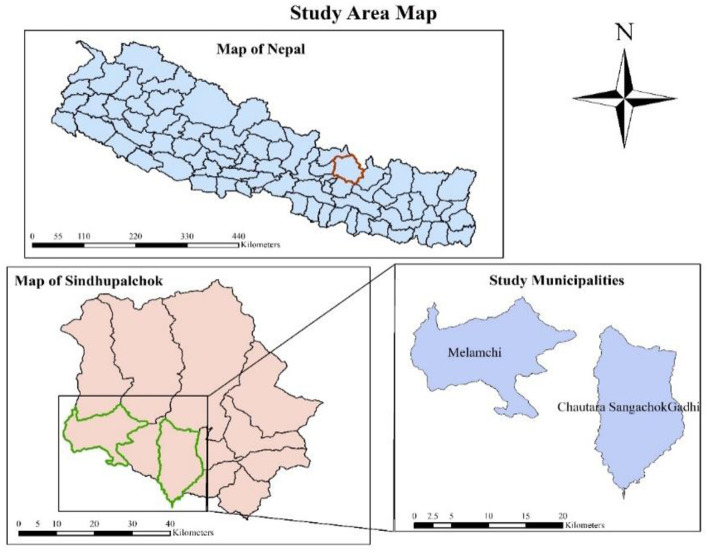
Location of the study site. Source: ArcGIS.

The research was conducted in the Sindhupalchok district. Sindhupalchok district is one of the districts of Bagmati province which is located at latitude 27° 46′ 0″ North, longitude 85° 42′ 0″ East. The district constitutes a total of three municipalities and nine rural municipalities. Out of this, Chautara Sangachowkgadhi and Melamchi municipalities were selected for the study as the plant science and animal science schools are located in these municipalities (Figure 1).

### 3.2 Participants

**Figure 2:**
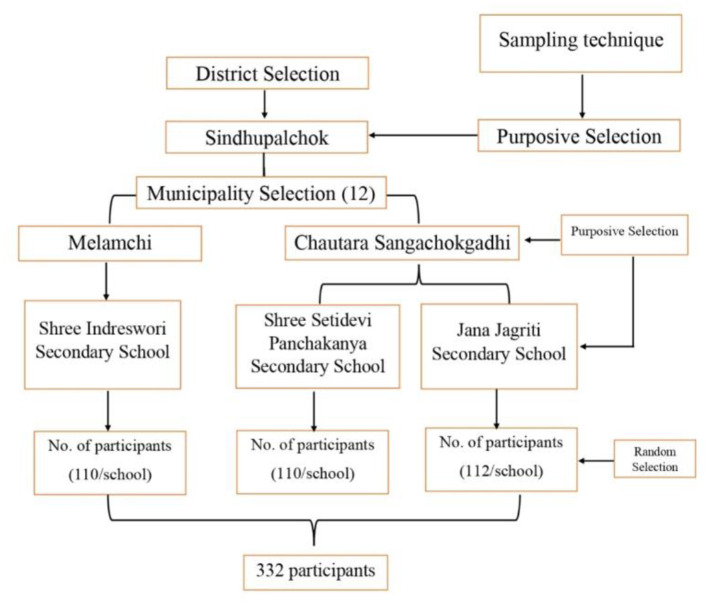
Schematic presentation of sample and sampling procedure.

The Ex-post-facto-research design with a deductive approach was used in this study. An ex-post-facto research design is a systematic empirical investigation in which independent variables have not been explicitly changed because they have already occurred or they are essentially non-manipulable (Pareek and JoShi, 2018). We conducted a cross-sectional survey of students who graduated from plant science and animal science programs (*n* = 332) in secondary schools in the Sindhupalchok district. Among the various districts of the Bagmati province, Sindhupalchok was specifically chosen since it is home to three TVE schools: two for plant science and one for animal science. The study was quantitative in nature and consisted of a 5-year census of secondary school graduates from 2018 to 2023 under the TVE program in the district. The TVE schools offering Plant Science and Animal Science are situated in the Melamchi and Chautara Sangachokgadhi municipalities. So, municipalities and secondary schools were selected purposively to ascertain career preferences from post-secondary agricultural graduates' viewpoint as a unit of assessment (Figure 2). On the other hand, in order to generalize the findings to a wider range of populations, the participants were chosen at random. The study was approved by the ethics committee of the Institute of Agriculture and Animal Science, Tribhuvan University, and informed consent was obtained from all respondents.

### 3.3 Questionnaire

The respondents were handed an 8-page entry questionnaire which was adapted and modified from the set of criteria developed by Takeda et al. (2013). The questionnaire mainly focused on sociodemographic questions, career preferences, and factors affecting career information. Sociodemographic information of respondents' age, gender, occupation, family size, family income source, interest in pursuing career in agriculture, and career counseling were included in the questionnaire. Additionally, the respondents were asked to choose their most preferred career: civil services (administration jobs), education and research institution jobs, agro-based public sector jobs, agro-based private sector jobs, self-employment/entrepreneurship, further education, and foreign employment. After choosing their preferred career, they were asked to answer to what degree these 28 items in the scale influenced the career preferences of TVE graduates. The factors affecting career preferences had 5 subscales namely personal (6 items), academic achievement (5 items), physical environment (5 items), social (6 items), and economic factors (6 items). A 5-point Likert scale (1 = Strongly Disagree, 2 = Disagree, 3 = Neutral, 4 = Agree, 5 = Strongly Agree) was used to rate the responses on the subscales.

### 3.4 Statistical analysis

The collected primary data was coded, entered, cleansed, and put forward for analysis. IBM SPSS Statistics−26 Version (Statistical Package for Social Science, Chicago, IL) and Microsoft Excel were used for the analysis of the obtained data. Descriptive statistics, including measures such as the mean, frequency, standard error of mean, and percentage, were computed to analyze the distribution of the variables in the study.

The factor analysis was conducted with IBM SPSS using a Maximum Likelihood method and Promax rotation with Kaiser Normalization. The parameter estimates in this method are determined in a way that maximizes the likelihood of obtaining the observed correlation matrix. Among 28 items in the questionnaire; 15 items were excluded due to low factor loading, Showing a similar factor loading (cross-loading) in more than two factors, and low communalities. Mean ± standard deviation (SD) was calculated for each item. Cronbach's alpha co-efficiencies demonstrated internal consistency i.e., scale reliability ranging between 0.72 and 0.85 and mean and standard deviation were also calculated.

## 4 Results

### 4.1 Respondents and career choice

The study examined the relationship between career preferences focusing on several sociodemographic characteristics such as gender, age, education level, household head occupation, family type, decision-making pattern, interest in pursuing a career in agriculture, and career counseling. The descriptions and comparisons were made based on the respondents' level of participation in interview schedules as illustrated in Table 1. Out of 166 respondents, 53.6% were males which shows the ratio of the target participants. Most of the respondents were under the age group of <20 (75.3%) with an educational level of class 12 (92.2%). The respondent's income source mainly includes agriculture (85.5%) and private services (9.6%). The majority of the respondents have a nuclear family (78.9%) and the father (55.4) as the decision-maker in the family. Among the total respondents, 56.7% and 69.9% of participants were interested in pursuing a career in agriculture and had career counseling respectively.

**Table 1:** Distribution of respondents based on sociodemographic characteristics.

**Socioeconomic characteristics (Sample size**, ***n*** = **166)**		
**Variable**	**Description of response**	**Frequency (%)**
Gender	Men	89 (53.6)
	Women	77 (46.4)
Age	<20	125 (75.3)
	21–25	27 (16.3)
	>25	14 (8.4)
Education level	Class 12	153 (92.2)
	Bachelor	13 (7.8)
Household head occupation	Agriculture	142 (85.5)
	Govt. service	8 (4.8)
	Private services	16 (9.6)
Family type	Joint	35 (21.1)
	Nuclear	131 (78.9)
Decision pattern	Father	92 (55.4)
	Mother	41 (24.7)
	Self	13 (7.8)
	Both	20 (12)
Interested in pursuing career in agriculture	Yes	94 (56.7)
	No	36 (21.6)
	Not decided yet	36 (21.6)
Career counseling	Yes	116 (69.9)
	No	50 (30.1)

### 4.2. Factor analysis

The pattern matrix contains the variable loading on each factor represented by a coefficient indicating the linear combinations of the items. The pattern matrix displayed in Table 2 showed that all items loaded exactly (values exceeding 0.40 on a single factor based on the sample size exceeding 150) on their hypothesized factors (Hair et al., 2009). The perfect item loading on their respective factors indicates adequate convergent validity. Only 5 factors were defined based on the items grouped together after factor analysis. Five factors have been identified that account for a combined 59.56% of the overall variation which is close to 60% as recommended (Shrestha, 2021). The calculated Cronbach's alpha ranged between 0.72 and 0.85 demonstrating internal consistency. The inter-factor correlation among the extracted factors is <0.80 indicating that factors do not largely share the same variance and have good discriminant validity (Kurbi et al., 2023) as mentioned in Table 2.

**Table 2:** Exploratory factor analysis associated with respondents career preferences.

**Items**	**Pattern (Factor loading)**				
	**1**	**2**	**3**	**4**	**5**
Factor 1: Academic achievement (Cronbach's α = 0.80)					
11) I consider my preferred career based on my academic achievements.	0.82	−0.13	−0.19	0.07	0.06
10) I consider my track in its grades in choosing a career.	0.82	−0.03	−0.22	0.05	0.14
9) I consider my previous grades in choosing my preferred career.	0.65	−0.07	−0.11	0.05	0.20
Factor 2: Physical environment (Cronbach's α = 0.73)					
12) I consider the place of my future work in choosing my career.	0.13	0.73	0.03	0.08	0.06
13) Far residency and/or inaccessibility to the desired workplace.	−0.01	0.64	0.17	−0.02	0.02
14) I consider the conducive learning environment of the workplace.	−0.19	0.61	0.09	0.05	0.02
16) I consider the quality of the workplace, where the job is being offered.	−0.25	0.54	−0.02	−0.07	−0.02
Factor 3: Social factor (Cronbach's α = 0.75)					
20) I consider the influence of my teacher in choosing my career.	−0.19	0.03	0.99	0.04	−0.07
22) My senior suggestion affects my career choice.	−0.22	0.18	0.67	−0.04	−0.09
Factor 4: Personal factor (Cronbach's α = 0.72)					
5) My preferred career is connected to my favorite subject.	0.07	−0.01	0.01	0.99	−0.04
4) My preferred career is my “childhood dream.”	0.04	0.04	−0.01	0.62	−0.06
Factor 5: Economic factor (Cronbach's α = 0.85)					
25) I consider my future employment in choosing my track.	0.20	0.06	−0.02	−0.02	0.99
28) I consider the job security.	0.08	−0.01	−0.13	−0.07	0.57
**Factor- Inter-factor correlation**	1	2	3	4	5
1	1.00				
2	−0.09	1.00			
3	−0.20	0.10	1.00		
4	0.08	0.02	0.02	1.00	
5	0.17	0.04	−0.06	−0.04	1.00

Maximum likelihood factor analysis and Promax rotation were conducted with IBM SPSS, which identified 5 factors associated with career preference based on a set of variables in a questionnaire conducted on post-secondary graduates under the TVE stream. Of the 28 items in the questionnaire, 15 were excluded because of the reasons described in Appendices.

### 4.3 Factors affecting career preferences of TVE graduates

In Table 3, factors associated with choosing Plant and Animal Science as a career among TVE post-secondary graduates are summarized based on the responses obtained from the respondents. The Likert mean scores are evaluated using the evaluation criteria presented below (Çelik and Oral, 2016), (Genç, 2017). Component 1, described as “Academic Achievement,” consists of three categories: previous grades, track in previous grades, and academic achievement. Among 3 categories, the previous grade of graduates showed a high level of agreement, as indicated by their mean score of 3.45. The other two elements, namely track in previous grades (3.00), and academic achievement (3.23), tend toward medium level of agreement based on their average score on the scale.

**Table 3:** Factors affecting career preferences of graduates from the TVE program.

**Factors**		**Sum**	**Mean**	**SEM**	**S.D**.	**Decision**
Items	Component 1: Academic achievement					
	I consider my previous grades in choosing my preferred career	572	3.45	0.09	1.12	High influence
	I consider my track in its grades in choosing a career	498	3.00	0.12	1.48	Medium influence
	I consider my preferred career based on my academic achievements	536	3.23	0.09	1.13	Medium influence
Items	Component 2: Physical environment					
	I consider the place of my future work in choosing my career	686	4.13	0.06	0.83	High influence
	Far residency a nd/or inaccessibility to the desired workplace	682	4.11	0.06	0.80	High influence
	I consider the conducive learning environment of the workplace	738	4.45	0.05	0.60	Very high influence
	I consider the quality of the workplace, where the job is being offered	700	4.22	0.06	0.81	Very high influence
Items	Component 3: Social					
	I consider the influence of my teacher in choosing my career	559	3.37	0.09	1.11	Medium influence
	My seniors' suggestion affects my career choice	550	3.31	0.10	1.25	Medium influence
Items	Component 4: Personal					
	My preferred career is my “childhood dream”	408	2.46	0.12	1.60	Low influence
	My preferred career is connected to my favorite subject	511	3.08	0.12	1.49	Medium influence
Items	Component 5: Economic					
	I consider my future employment in choosing my track	662	3.99	0.05	0.66	High influence
	I consider the job security	697	4.20	0.06	0.81	Very high influence

SEM, Standard error of mean; S.D., Standard deviation.

In component 2- Physical environment the item conducive learning environment in the workplace has the highest mean score of 4.45, indicating a very high influence on the career preference of graduates followed by the item quality of the workplace (4.22). On the other hand, the items place of future work (4.13), and far residency (4.11) tend to have a high level of agreement, as indicated by their mean scores on the scale in Table 3. Component 3 named Social comprised two items and they were the influence of teachers' and seniors' suggestions. The mean score for the influence of teachers (3.37) and seniors' suggestions (3.31) indicates a medium-level tendency toward agreement for career preferences.

Component 4 was labeled Personal in which the item's favorite subject had a maximum mean score of 3.08 which inclined toward a medium level of agreement, whereas childhood dream had a mean score of 2.86 which tends to have a low level of influence on the career preference of post-secondary graduates under the TVE program (Table 3). Similarly, in Component 5, job security has the highest mean score of 4.20 which indicates a very high level of influence while future employment has a mean score of 3.99 which implies a high level of influence.

## 5 Discussion

Dhungel et al. (2017) found that a higher proportion of male students were enrolled in surveyed schools under the TVE program compared to female students. The greater number of male respondents may be attributed to several variables, such as individual interests and preferences, parental influences, school-related factors, and teacher-related factors (Tanga and Mushoriwa, 2019). Regarding the household head occupation, one of the reasons could be that the majority of the interviewed population lacks additional life skills beyond farming (Tittonell et al., 2010). The expertise beyond agriculture is crucial for improving one's standard of living (Collier and Dercon, 2014). In the agricultural setting, individuals possessing diverse life skills can enhance family economies (Dulal, 2019).

In this study, families were classified as either nuclear families, which consisted of a husband, wife, and their unmarried children, or joint families, which included a husband, wife, children, and their in-laws living together. The result demonstrated that individuals residing in nuclear families exhibited greater engagement in farming and their associated activities (Raut, 2018). In addition, most of the decision-making was done by the father compared to the mother which may be due majority of the households being men-headed. This result also aligns with the findings of Pandey (2021) and Fu and Deshpande (2014). Inadequate knowledge, passion, and interest have a great influence on the career preferences of high school students (Aschbacher et al., 2010). Additionally, implied that earlier career guidance and counseling of high school students can provide them with insights to select better careers (Perdrix et al., 2012).

An Exploratory Factor Analysis (EFA) was conducted to study the data on the factors that influence the career preferences of agricultural students from secondary schools in the TVE program. The purpose of the analysis was to find the latent factor structure within the data. The EFA determined that the Kaiser-Meyer-Olkin (KMO) value of 0.590 was above the minimal threshold of 0.500 (Na-Nan et al., 2020). Bartlett's test of sphericity was used to assess the suitability of the correlation matrix. Bartlett's test yields a highly significant result at a significance level of *p* < 0.001. This indicates that the correlation matrix exhibits substantial correlations among at least a subset of the variables (Stevens, 2002). Bartlett's Chi-square was calculated which is χ2 = 619.447 (*p* < 0.01). Therefore, the hypothesis that the correlation matrix is an identity matrix is disproven. More precisely, the variables are not orthogonal to one other. A *p*-value <0.05 suggests that conducting a factor analysis on the data set may be beneficial (Rouquette and Falissard, 2011).

Academic achievement is often viewed as an indicator of employees' work ethics and commitment (Mustofa et al., 2022; Fu and Deshpande, 2014). Moreover, having higher grades in secondary school could help to compete with other employees. The results coincide with the research findings (Quadlin, 2018) that argue employers highly regard the competence and dedication exhibited by male candidates. Blotnicky et al. (2018) indicated in their study that students with average track in previous grades and academic achievement have limited knowledge with respect to their career and their preferences.

A conducive learning environment in the workplace helps to promote skills and adopt a passion for the respective field (Noe et al., 2014; Chandrasekar, 2011). Siew et al. (2015) reported a similar argument that indicates Engaging in the hands-on workshop activity stimulated reflection on strategies to foster greater creativity and a genuine enthusiasm for the profession. Quality of the workplace includes factors such as rewards, recognition, and workplace culture which greatly encourages employees to grow (Ali and Anwar, 2021; Anitha, 2014). The provision of a high-quality workplace that satisfies the intellectual and emotional needs of its members empowers them to maximize their abilities and fosters constructive collaboration, ultimately leading to enhanced performance (Joshi, 2016; Chew and Mohamed Zainal, 2024). Similarly, place of future and far residency also has a high influence on career preference as the students may choose a workplace known for a specific industry or prioritize jobs near their hometown (Monk et al., 2012; Ceric and Crawford, 2016). A similar finding about the strong desire of employees to select jobs closer to their hometown (Engel and Cannata, 2015).

The influence of teachers and advice from seniors influences the career preferences of graduates. This might be because they can encourage, motivate, and guide them through their experience and mentorship (Ng et al., 2021; Austin, 2002). Montgomery et al. (2014) revealed that Exemplary seniors and teachers have a direct impact on personal development and greater commitment to their careers of students in a variety of fields. A subject that a student finds engaging has the potential to provide them with skill development and expertise, ultimately leading them to pursue a profession in which they specialize (Chu et al., 2021). Similar findings regarding the attempt to develop disciplinary literacy in students through the motivation of their preferred subject matter, with the ultimate goal of involving them in a chosen profession (Shanahan and Shanahan, 2012). However, childhood dreams had a comparatively lower influence on graduates' career preferences, which might be due to the experience gained, exposure to careers, and their interests (Nungsari et al., 2024; Mortimer et al., 2002). Children might gain confidence in their chosen profession as a result of the novel experiences they encounter (Chen et al., 2023; Lasky, 2005). This might be because students focus on careers with high demand, financial stability, and long-term careers (Pignault et al., 2022). According to Ko and Jun (2015), graduates emphasize motivating factors such as future employment, financial stability, and job security, in contrast to other aspects.

## 6 Limitations

The limitations identified for the study were time and resource constraints, generalizability, non-response bias, Hawthorne effect, influences outside the scope of the research, and lack of longitudinal data. The research is unable to cover all areas and limits itself to the local-level study of a few municipalities and rural municipalities of the Sindhupalchok district. The samples in this research were small in size (*n* = 332) and had to be confined to sampled high school agricultural graduates of Sindhupalchok district because of the limited time and resources. Due to the reliance on written responses from respondents, it was not possible to eliminate the potential for bias in the data. It is also likely that certain responses may not accurately or completely reflect the career preferences of the respondents (Kock et al., 2021). However, it is anticipated that the study will provide insight into the career preferences of TVE graduates. The Hawthorne Effect presented a challenge to the validity of this study. Participants may have offered responses that they perceived as socially desirable (Jiménez-Buedo, 2021). One constraint of this aspect of the study was the potential for students to alter their intentions as time progressed (Hossain et al., 2023).

## 7 Conclusion

The present study indicated that agriculture students possess a positive and practical view of career choice. Nonetheless, the majority of post-secondary graduates expressed a preference for overseas employment. They emphasized the significance of the following considerations when selecting a future profession: “conducive learning environment,” “quality of work” and “job security,” while “childhood dream” and “favorite subject” did not seem important. Furthermore, this study suggested that the significance of “job security” and “quality of work” in the agricultural sector is far greater than advocated by researchers in agriculture as a means to choose a career. Engaging students in early career development could enhance their passion for more rewarding career options and increased job satisfaction. The establishment of a plant science stream and animal science stream in secondary schools is anticipated to enhance the nation's human resource capacity to advance agriculture development in Nepal.

## Data Availability

The original contributions presented in the study are included in the article/Supplementary material, further inquiries can be directed to the corresponding author.
