# Supplementary material for: Factors associated with the choice of plant and animal science as a career among technical and vocational education post-secondary graduates in Nepal
Source: Front Sociol. 2025 Sep 2;10:1585391. doi: 10.3389/fsoc.2025.1585391 (PMC12436333; doi:10.3389/fsoc.2025.1585391)
Supplement: Supplementary file 1 [file Data_Sheet_1.pdf]

Factors affecting career preferences of graduates from the TVE program

These 28 items were used to analyze the factors affecting career preference of graduates from the TVE program. The items were categorized into six groups as following:

Items

Personal:

1. My choice of school affects my choice of career.
2. My preference for a career is suited to my talent.
3. My preference of career is my personal choice.
4. My preferred career is my “childhood dream”.
5. My preferred career is connected to my favorite subject.
6. My preferred career is an in-demand career.

Academic qualification:

7. I consider the academic program based on internship opportunities and hands-on experiences.
8. I consider my present scholarship for it is an automatic qualification to enroll in my choice of career.
9. I consider my previous grades in choosing my preferred career.
10. I consider my track in its grades in choosing a career.
11. I consider my preferred career based on my academic achievements.

Physical Environment:

12. I consider the place of my future work in choosing my career.
13. Far residency and/or inaccessibility to the desired workplace.
14. I consider the conducive learning environment of the workplace.
15. I consider the uniform of the workplace in choosing a career.
16. I consider the quality of the workplace, where the job is being offered.

Social Factor:

17. My friend’s preference for a career affects my career choice.
18. My parents are the ones who are choosing my career.
19. I consider the dominant profession of my family in choosing my career choice.
20. I consider the influence of my teacher in choosing my career.
21. I consider the choice of my sponsor in choosing my career.
22. My senior suggestion affects my career choice.

Economic Factor:

23. I consider the financial status of my family.
24. I consider my supposed future earnings.
25. I consider my future employment in choosing my track.
26. I consider my preferred track for I could already earn an income while studying.
27. I consider the cost/expenses in choosing a course.
28. I consider the job security.

Out of 28 items, 15 items were excluded from factor analysis because of

Low factor loading(<0.5)

3. My career preference is my personal choice.
18. My parents are the ones who are choosing my career.
19. I consider the dominant profession of my family in choosing my career choice.
21. I consider the choice of my sponsor in choosing my career.
24. I consider my supposed future earnings.
26. I consider my preferred track for I could already earn an income while studying.
27. I consider the cost/expenses in choosing a course.

Showing a similar factor loading (cross-loading) in more than two factors

7. I consider the academic program based on internship opportunities and hands-on experiences.

15. I consider the uniform of the workplace in choosing a career.

17. My friend's preference for a career affects my career choice.

Low communalities (<0.5)

1. My choice of school affects my choice of career.

2. My preference for a career is suited to my talent.

6. My preferred career is an in-demand career.

8. I consider my present scholarship for it is an automatic qualification to enroll in my choice of career.

23. I consider the financial status of my family.

Table 1: Exploratory factor analysis associated with respondents' career preferences (Pattern matrix)

Items	Pattern Matrix (Factor loading)				
	1	2	3	4	5
1. My choice of school affects my choice of career.	-.041	.019	.112	-.100	.405
2. My preference of career is suited to my talent.	.057	.260	-.059	-.026	.486
3. My preference of career is my personal choice.	-.018	-.033	-.138	-.131	.297
4. My preferred career is my "childhood dream".	-.076	-.006	.054	.015	.849
5. My preferred career is connected to my favorite subject.	-.067	.015	.020	.033	.718
6. My preferred career is an in-demand career.	.213	-.243	-.055	.064	.503
7. I consider the academic program based on internship opportunities and hands-on experiences.	.427	.010	.058	-.008	.111
8. I consider my present scholarship for it is an automatic qualification to enroll in my choice of career.	.106	.463	-.044	.065	-.018
9. I consider my previous grades in choosing my preferred career.	.105	.635	.001	.024	.029

10. I consider my track in its grades in choosing a career.	-.015	.813	.072	-.084	-.033
11. I consider my preferred career based on my academic achievements.	-.073	.827	-.056	-.030	-.017
12. I consider the place of my future work in choosing my career.	.026	.199	.630	.000	.085
13. Far residency and/or inaccessibility to the desired workplace.	.019	.109	.624	.101	.013
14. I consider the conducive learning environment of the workplace.	.043	-.085	.614	-.038	.007
15. I consider the uniform of the workplace in choosing a career.	-.004	-.080	.600	.040	-.069
16. I consider the quality of the workplace, where the job is being offered.	.035	-.193	.617	-.195	-.006
17. My friend's preference for a career affects my career choice.	.023	.018	-.081	.576	.018
18. My parents are the ones who are choosing my career.	.037	-.203	-.188	.034	-.068
19. I consider the dominant profession of my family in choosing my career choice.	.006	.119	-.111	.235	-.104
20. I consider the influence of my teacher in choosing my career.	-.053	.011	.053	.882	-.018
21. I consider the choice of my sponsor in choosing my career.	.068	-.194	-.166	.501	.065
22. My seniors' suggestion affects my career choice.	-.061	-.004	.185	.698	-.098
23. I consider the financial status of my family.	.543	.133	-.052	.021	-.017
24. I consider my supposed future earnings.	.851	.064	.029	-.011	-.058

25. I consider my future employment in choosing my track.	.926	.037	.004	.021	.022
26. I consider my preferred track for I could already earn an income while studying.	.471	.040	.079	.197	.039
27. I consider the cost/expenses in choosing a course.	.380	-.127	.083	-.108	-.088
28. I consider the job security.	.626	-.061	-.053	-.109	-.060

Appendix 2: Communalities of factors affecting career preferences of graduates from the TVE program

Items	Initial	Extraction
1. My choice of school affects my choice of career.	.316	.182
2. My preference of career is suited to my talent.	.386	.354
3. My preference of career is my personal choice.	.226	.128
4. My preferred career is my “childhood dream”.	.578	.722
5. My preferred career is connected to my favorite subject.	.543	.520
6. My preferred career is an in-demand career.	.429	.332
7. I consider the academic program based on internship opportunities and hands-on experiences.	.381	.201
8. I consider my present scholarship for it is an automatic qualification to enroll in my choice of career.	.344	.241
9. I consider my previous grades in choosing my preferred career.	.434	.436
10. I consider my track in its grades in choosing a career.	.598	.672
11. I consider my preferred career based on my academic achievements.	.618	.696
12. I consider the place of my future work in choosing my career.	.434	.400
13. Far residency and/or inaccessibility to the desired workplace.	.388	.394
14. I consider the conducive learning environment of the workplace.	.425	.399
15. I consider the uniform of the workplace in choosing a career.	.382	.400
16. I consider the quality of the workplace, where the job is being offered.	.467	.463
17. My friend’s preference for a career affects my career choice.	.546	.329
18. My parents are the ones who are choosing my career.	.506	.071
19. I consider the dominant profession of my family in choosing my career choice.	.458	.078
20. I consider the influence of my teacher in choosing my career.	.642	.786
21. I consider the choice of my sponsor in choosing my career.	.576	.335

22. My seniors' suggestion affects my career choice.	.550	.557
23. I consider the financial status of my family.	.474	.341
24. I consider my supposed future earnings.	.754	.748
25. I consider my future employment in choosing my track.	.776	.872
26. I consider my preferred track for I could already earn an income while studying.	.432	.277
27. I consider the cost/expenses in choosing a course.	.355	.169
28. I consider the job security.	.532	.396

After excluding 15 items, the communalities of the remaining items are presented in Appendix 3.

Appendix 3: Results of communalities for factors affecting career preferences

Communalities		
	Initial	Extraction
My preferred career is my "childhood dream".	.402	.486
My preferred career is connected to my favorite subject.	.404	.898
I consider my previous grades in choosing my preferred career.	.377	.431
I consider my track in its grades in choosing a career.	.546	.670
I consider my preferred career based on my academic achievements.	.542	.686
I consider the place of my future work in choosing my career.	.399	.574
Far residency and/or inaccessibility to the desired workplace.	.324	.420
I consider the conducive learning environment of the workplace.	.363	.493
I consider the quality of the workplace, where the job is being offered.	.343	.445
I consider the influence of my teacher in choosing my career.	.519	.809
My senior suggestion affects my career choice.	.513	.506
I consider my future employment in choosing my track.	.399	.845
I consider the job security.	.382	.535
